# Moderate Beer Intake Downregulates Inflammasome Pathway Gene Expression in Human Macrophages

**DOI:** 10.3390/biology10111159

**Published:** 2021-11-09

**Authors:** Natàlia Muñoz-Garcia, Rafael Escate, Lina Badimon, Teresa Padro

**Affiliations:** 1 Cardiovascular Program-ICCC, Research Institute—Hospital Santa Creu i Sant Pau, IIB-Sant Pau, 08025 Barcelona, Spain; nmunoz@santpau.cat (N.M.-G.); rescate@santpau.cat (R.E.); tpadro@santpau.cat (T.P.); 2 Centro de Investigación Biomédica en Red Cardiovascular (CIBERCV), Instituto de Salud Carlos III, 28029 Madrid, Spain; 3 Cardiovascular Research Chair, UAB, 08193 Barcelona, Spain

**Keywords:** inflammasome, beer, macrophages, atherosclerosis, gene expression, fermented beverages, cardiovascular disease, priming, activation, interleukins

## Abstract

**Simple Summary:**

Moderate consumption of fermented beverages is associated with prevention against diseases involving inflammation and immunity. Conversely, for high drinking levels, an increase of inflammatory mediators and the susceptibility to infections occur, which tend to offset the benefits in terms of health. Unfortunately, this area remains poorly understood. Inflammation is now recognized as an overwhelming burden for public health. Thus, subcellular molecular complexes such as the “inflammasomes” have been identified as key players in cellular stress and tissue damage, and as regulators of immune and inflammatory responses. Here, we investigated the impact of moderate intake of alcohol-free and traditional beer in humans on the inflammasome pathway of activated pro-inflammatory human macrophages. The results of this study showed that macrophages submitted to a pro-inflammatory stimulus to activate the inflammosome had a mitigated inflammatory response in the presence of blood serum obtained from healthy volunteers after consuming alcohol-free or traditional beer for four weeks (women one can and men two cans per day) compared to the response found in the presence of blood serum obtained before beer intake. This was shown by a decrease in end components of the inflammasome cascade (IL-1β and TNF) at gene expression and protein level as found with alcohol-free and traditional beer, respectively.

**Abstract:**

Inflammasomes are key components of the innate immunity system that trigger the inflammatory response. Inappropriate activity of the inflammasome system has been linked to onset and perpetuation of inflammation in atherosclerotic plaques and cardiovascular disease. Low-to-moderate beer consumption is inversely associated with cardiovascular event presentation, while high levels of alcohol intake are associated with increased cardiovascular risk. Although fermented beverages have been suggested to exert their beneficial effects through their anti-oxidant and anti-inflammatory properties, little is known regarding the capacity of beer to modulate innate immunity cell responses. To this aim, primed or activated THP-1 macrophages were conditioned with human serum obtained from a prospective two-arms longitudinal crossover study to investigate the effect of a moderate and regular daily intake of beer, either alcohol-free or traditional, in the regulation of TLR-mediated inflammatory responses in healthy but overweight individuals. Conditioned macrophages with serum obtained after four-week intervention with alcohol-free beer significantly reduced the transcription of pro-inflammatory interleukins such as IL-1β and TNF. The serum of traditional beer consumers did not exhibit the same capacity as the serum of alcohol-free beer consumers to reduce gene expression of pro-inflammatory interleukins; however, serum from traditional beer consumers showed a regulatory effect at the protein level by significantly decreasing the intracellular protein levels of pro-IL-1β in primed macrophages and preventing cleaved-IL-1β protein release.

## 1. Introduction

Adverse innate immune responses are a critical component in several disease processes, including cardiovascular disease (CVD). Atherosclerosis is characterized by continuous inflammatory process in the arterial wall in which monocytes and macrophages play a crucial role. Thus, macrophages, differentiated from circulating monocytes, participate in the processes of innate immunity and express sensors for both exogenous and endogenous “danger” signals. The subcellular molecular structures called “inflammasomes” are key components to the defense of the cell against stress and cell/tissue damage and regulators of immune and inflammatory responses. Inappropriate activity of the inflammasome system has been linked to onset or progression of inflammation in atherosclerotic cardiovascular disease [1].

The inflammasome activation pathway includes the family of transmembrane Toll-like receptors (TLR), RIG-1-like helicases (RLR), and cytoplasmic sensors such as AIM2-like receptors (ALRs) and nucleotide-binding domain and leucine-rich repeat-containing family receptors (NLRs). They are all involved in the recognition, by immune cells, of molecular patterns associated with pathogens (PAMP) or molecular patterns associated with intracellular and extracellular damage (DAMP) [1]. Several members of the NLR family such as NLRP1, NLRP3, and NLRC4 are assembled into large multiprotein complexes called inflammasomes that induce the activation of pro-caspase-1 into caspase-1, which in turn, cleaves the pro-inflammatory cytokines pro-IL-1 and pro-IL-18 in the mature forms (IL-1 and IL-18). Inflammasomes are, therefore, multimeric protein complexes that, after activation, promote the generation of biologically active cytokines [2]. The induction of inflammasome pathways via lipopolysaccharide (LPS) also induces IL-6 and TNF secretion [3,4], and it is also suggested that caspase-8 is required for both the transcriptional priming and activation of the canonical and non-canonical NLRP3 inflammasome pathways [5,6].

The increase or aberrant expression of cytokines involved in the inflammasome signalling has been related to different immune and inflammatory pathologies as well as cancer and cardiovascular diseases [7,8,9,10,11]. As such, there is a growing interest in gaining better understanding in the inflammasome as a new therapeutic target in cardiovascular disease.

Alcohol consumption may induce changes in both innate and adaptive immunity [12]. The beneficial or detrimental effects of alcohol in cardiovascular health seem to relate to a dose response pattern. While low to moderate alcohol intake has been related to a low risk of CVD mortality, excessive alcohol consumption has been linked to deleterious effects on health [13]. The benefits of moderate alcohol intake have been associated with increased levels of high-density lipoprotein (HDL), decreased low-density lipoprotein (LDL) levels, reduced platelet aggregation, anti-atherogenic and anti-thrombotic effects, and decreased inflammation [14,15].

Alcoholic beverages are not all equal in composition; fermented beverages are thought to provide better cardiovascular protection than spirits due to the beneficial effects of their polyphenolic content, which has been described to exhibit anti-inflammatory, anti-oxidant, and anti-aggregation effects [16].

Polyphenols represent a large group of secondary metabolites synthesized by plants. Studies with isolated polyphenols have been described to interfere with pro-inflammatory pathways, such as the NLRP-3 inflammasome, the nuclear factor kappa β (NF-kβ), and the mitogen-activated protein kinase (MAPK). In particular, in vitro studies with these compounds have suggested that polyphenol-induced inhibition of these transcription factors or macromolecular complexes may reduce the synthesis and release of pro-inflammatory cytokines such as interleukin IL-1β, IL-6, IL-8, and the factor of tumor necrosis (TNF), which in turn have been associated with adverse CV events [17]. In addition, polyphenolic compounds have been shown capable of attenuating the inflammasome by inhibiting the priming signal [18].

Not only the polyphenolic compounds in vitro have shown these effects, but metabolites derived from alcohol hydrolysis have also been preliminary shown to specifically inhibit the NLRP3 inflammasome [19]. In vitro studies on human monocytes isolated from peripheral blood mononuclear cells (PBMCs) showed that pre-incubation for 24 h with 25 mM alcohol (≈0.1 g/dL blood alcohol concentration) inhibits nuclear translocation of NF-κB in response to LPS, and thereby production of pro-inflammatory cytokines [20]. Similarly, a study conducted on stimulated RAW 264.7b (macrophages-like), demonstrated a decreased TNF production after cell treatment with 25 mM of ethanol associated with an increased expression of IL-1R-associated kinase-monocyte (IRAK-M), a negative regulator of LPS signalling [21]. However, there is no information on the effects over the inflammasome of moderate beer intake in humans.

In a previous intervention study in healthy obese and overweight volunteers, we demonstrated that moderation of both traditional and alcohol-free beer did not have any harmful vascular effects, did not increase weight and increased the anti-oxidant capacity of HDL as well as the capacity to promote cholesterol efflux, which may prevent lipid deposition in the vascular wall [22]. Up to now, however, available results on the effects of fermented alcohol beverages on the immune system are inconclusive and those focusing on alcohol consumption have mostly been derived in apparently controversial findings [12,23].

The differences in the pathologic status associated with obesity or overweight might condition inflammasome responses. An increased BMI has been linked to metabolic alterations including oxidative stress and inflammation that in turn may contribute to inflammasome activation. Therefore, we investigated in a population of obese and overweight individuals whether regular and moderate beer consumption modulates the functional behavior of human macrophages when exposed to external pro-inflammatory stimulus. Our findings proved that human macrophages (THP-1 derived), conditioned with blood serum from healthy subjects after four weeks of moderate daily intake of beer (traditional and alcohol-free beer), depicted a switch to a lower responsiveness of the inflammasome pathway after being exposed to the bacterial cell wall component lipopolysaccharide (LPS).

## 2. Materials and Methods

### 2.1. Study Population

Thirty-six healthy adult individuals between ages of 40–60 years, non-smokers, regular but moderate beer consumers (self-reported consumption), and with overweight (body mass index (BMI) of 28–29.9 kg/m^2^) or obesity class 1 (BMI of 30–35 kg/m^2^) were recruited for the study. All subjects were randomly subjected to two 4-week treatment sequences (alcohol free or traditional beer), separated by a 4-week wash-out period as previously described [22]. Briefly, men drank two cans (660 mL beer) and women one can (330 mL beer) per day of alcohol-free beer (0.0 g alcohol and 414 mg polyphenols/can) or traditional beer (15 g of ethanol, equivalent to 5.7% Vol and 604 mg polyphenols/can) during the intervention periods. Participants abstained from drinking alcoholic beverages and alcohol-free beer out of those provided as part of the study both during the run-in and wash-out periods and throughout the intervention phases. Traditional and alcohol-free beers were of the lager type from the same Spanish commercial brand. They presented similar phenolic composition although differing in the content of the different components [22].

Moderate beer drinking was defined according to the “Dietary Guidelines for Americans 2015–2020,” U.S. Department of Health and Human Services and U.S. Department of Agriculture, (https://www.niaaa.nih.gov/alcohol-health/, accessed on 5 November 2021, overview-alcohol-consumption/moderate-binge-drinking) and refers up to 1 drink per day for women, and up to 2 drinks per day for men. The study complies with the Declaration of Helsinki and was approved by the Human Ethical Review Committee of the Hospital “Santa Creu i Sant Pau” of Barcelona (Ref 14/186; 12 November 2014). Informed written consent was obtained from all participants before entering the study.

### 2.2. Experimental Design

**Figure 1:**
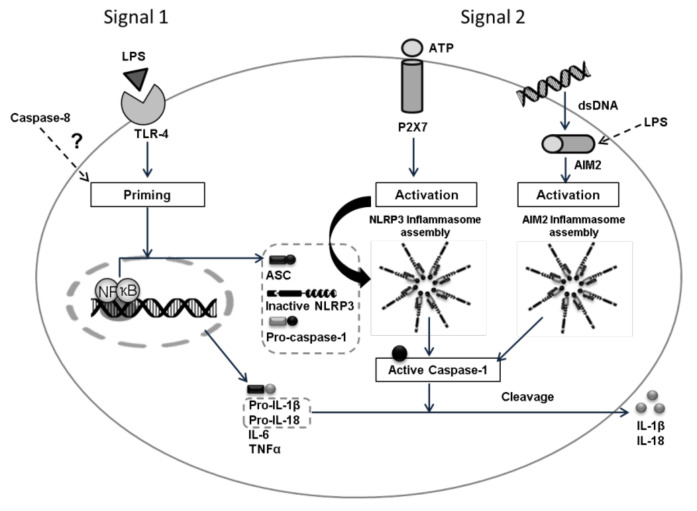
Activation of the inflammasome requires two signals: Signal 1 (priming) is triggered by the recognition of PAMPs/DAMPs (i.e., LPS) by PRRs (Pattern Recognition receptors), such as Toll-like receptors (TLR) and drives the nuclear translocation of NF-κB and the transcription of its target genes. For the activation, signal 2 induces the assembly of NLRP3 (i.e., ATP) or AIM2 (i.e., dsDNA), ASC, and Caspase-1 supracomplexes to form an active NLRP3 or AIM2 inflammasome, where active Caspase-1 processes pro-IL-1β/pro-IL-18 into IL-1β/IL-18 mature forms that will be secreted into the extracellular space [1]. The role of caspase-8 remains unclear, but it is thought that it has an important role regulating the inflammasome priming pathway [5].

The study has been performed in human macrophages differentiated from the monocytic cell line THP-1 by PMA (Phorbol 12-myristate 13-acetate). The inflammasome pathways (priming and activation) were investigated on macrophages after conditioning with human serum obtained before and after beer interventions. To assess the cellular response, different molecular effectors or intermediaries such as: Caspase-8, IL-6, TNF, IL-18, and IL-1β were analyzed (Figure 1). Briefly, macrophages were treated for 4 h with 100 ng/mL LPS (signal 1) in order to stimulate the expression of the cellular inflammasome (priming) and human serum (SH) obtained from each volunteer before and after the intervention with traditional or alcohol-free beer. In a second study, macrophages were treated with 5 mM ATP (signal 2) during the last 30 min of incubation with LPS (100 ng/mL, signal 1) in order to facilitate the activation of the inflammasome system. After the induction/activation period, cells were collected in the corresponding lysis buffer to extract the RNA and protein by means of standardized procedures that allow their subsequent quantitative analysis. The supernatants (SN) were collected individually (*n* = 36, 4 conditions: blood withdrawal before and after each beer intervention) and frozen at −80 °C for subsequent analysis of the cellular secretome.

### 2.3. Human Serum

The human serum used to condition the macrophages and investigate the regulation of the cellular inflammasome was collected during the previously described clinical study and preserved at the ICCC sample repository [22]. Blood samples obtained in this study were collected after twelve hours of fasting. Plasma and serum fractions were collected after centrifugation at 1800× *g* for 20 min and stored at −80 °C. Serum from 36 volunteers before and after 4 weeks of regular consumption in moderate amounts of traditional beer or alcohol-free beer was investigated (Figure S1).

### 2.4. Cell Cultures

**Figure 2:**
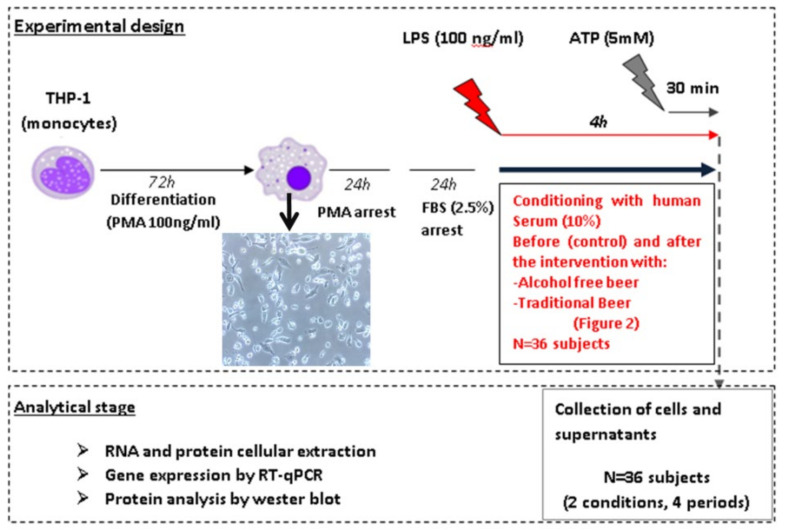
Summary scheme of the experimental design study of the response of the inflammasome profile in cells incubated in the presence of human serum. The human serum-induced effect of overweight (BMI: 28.0–29.9 kg/m^2^) or obese (BMI: 30.0–35.0 kg/m^2^) individuals was investigated before and after the traditional and alcohol-free beer interventions on the expression of different molecular components of the inflammasome, at the mRNA level, by real-time quantitative PCR (gene mRNA expression), and at the protein level in cell lysates and culture media (secretome), based on the Western blot technique (WB) and specific immunoassays (ELISA).

Human THP-1 monocytes were cultured in RPMI culture media supplemented with 10% FBS, 1% HEPES, 1% L-Glutamine, and 1% penicillin/streptomycin and differentiated to macrophage through the activation of the NF-kβ pathway by PMA (Sigma Aldrich, St. Louis, MO, USA). To this aim, 500,000 cells/well were seeded in 12-well plates and treated with PMA at a final concentration of 100 ng/mL for 72 h. Once differentiated, macrophages were subjected to 2 successive washing periods of 24 h each, to eliminate the effects of PMA (RPMI media supplemented with 10% FBS, 1% HEPES buffer, 1% L-glutamine and 1% P/S) and synchronize the gene expression of the cells before starting the stimulation (RPMI supplemented with 2.5% FBS, 1% HEPES buffer, 1% L-glutamine and 1% of P/S) with the human serum and activators (Figure 2). Gene expression levels of different markers from differentiated macrophages were determined by RT-qPCR in order to discriminate macrophages from the monocytic origin (Figure S2).

The cellular inflammasome pathway was induced by priming the macrophages with LPS (100 ng/mL) during 4 h in the presence of the conditioning human serums (10%) from the different intervention periods. Additionally, inflammasome activation was triggered by incubating the cells with LPS (100 ng/mL) during 4 h in the presence of human serum (10%) and adding ATP (5 mM) in the last 30 min (see Figure 1 and Figure 2).

### 2.5. Real-Time PCR Analysis

Total RNA from macrophages was extracted using the RNeasy Mini Kit (Qiagen) according to manufacturer’s instructions. RNA concentration was determined with a NanoDrop ND-1000 spectrophotometer (NanoDrop Technologies; Thermo Fisher Scientific; Waltham, MA, USA), and purity was assessed by the A260/A280 ratio. Reverse transcription of genes was performed with the High Capacity cDNA Reverse Transcription Kit followed by Taqman real-time PCR amplification, according to the manufacturer’s instruction (Applied Biosystems). Gene expression levels were analyzed by specific primers (ThermoFisher Scientific, Waltham, MA, USA) for IL-1β (Hs01555410_m1), IL-18 (Hs01038788_m1), IL-6 (Hs00174131_m1), TNF (Hs00174128_m1), Caspase-8 (Hs01022438_m1), NF-κB (Hs00231653_m1), NLRP3 (Hs00918082_m1) and AIM2 (Hs00175457_m1). For the characterization of the THP-1 macrophages, the gene expression levels of the following markers were analyzed: CD4 (Hs01058407_m1), CD11c (Hs00174217_m1), CD49c (Hs00233722_m1), CD14 (Hs00169122-g1) and CD16 (Hs00275547_m1). Samples were analyzed in duplicates, and only mRNAs with expression levels below 32 cycles were accepted. PIK3C2A (Hs00153223_m1) was used as endogenous internal control to normalize the mRNA expression levels. Further primer information is depicted in Table S1. Data were analyzed by SDS 2.4, RQ Man-ager 1.2.1 and DataAssist v3.0.1 software (Applied Biosystems, Foster City, CA, USA).

### 2.6. Western Blot Analysis

To prepare the cell lysates, cells were extracted in RIPA buffer (PH 8) followed by sonication to disrupt the cellular membranes and centrifugation (10,000× *g*, 7 min at 4 °C) to remove the cellular debris. A total amount of protein of 2 µg was used to perform the analysis.

Cellular supernatants (1 mL) were centrifuged (1200× *g*, 10 min at 4 °C) to remove the remaining cells, concentrated an average of 4–5 fold times in Amicon Ultra-4 kgl filter units (Merck Milipore, Burlington, MA, USA) and depleted for albumin/IgGs with the ProteoExtract Albumin/IgG removal kit (Merck Millipore, Burlington, MA, USA).

Sample extracts from the cellular/supernatant fraction were resolved by SDS-PAGE and electro-transferred to nitrocellulose membranes. Detection of the protein of interest was performed with antibodies against total IL-1β (monoclonal anti-IL-1β, Cell Signaling, 1:1000) and cleaved IL-1β (monoclonal anti-IL-1β, Cell Signaling, 1:1000) and normalized with β-actin (monoclonal anti-β-actin, Abcam, dilution:1/5000) or total protein (ponceau). Western blot bands were detected by chemiluminescence using a peroxidase enzymatic reaction (Supersignal, Pierce) and quantified with a ChemiDocTM XRS system using the Quantity-One 1-D analysis software (Bio-Rad).

### 2.7. Statistical Analyses

Statistical analyses were conducted using StatView 5.0.1 software (SAS Institute, Cary, NC, USA) and SPSS software (IBM SPSS Statistics 25.0.0, New York, NY, USA) except when indicated. Data are expressed by the number of cases (qualitative variable) and as mean ± SEM or median [IQR] for the quantitative variable. For all analyzed variables, values at the end of the run-in and the wash-out period were considered as the baseline value for the following intervention period. The normal distribution of variables was analysed by the Kolmogorov–Smirnov test. Differences in the baseline characteristics and in the percentage of change between intervention-diets were analyzed by unpaired Student’s *t*-test for parametric variables and Mann–Whitney test for non-parametric variables. When needed, Chi-square analysis was performed as indicated in the Results section. Effects of the 4-week interventions were evaluated using a paired Student’s *t*-test (baseline and post-intervention values) or Wilcoxon when normality failed. When considered, repeated measures ANOVA test was performed for paired samples as well as an analysis of variance (ANOVA) test introducing the different obesity and lipid-related variables as co-variable when required. A Bonferroni post-hoc test was run for two group comparisons after ANOVA. All reported *p*-values are two-sided, and a *p*-value of 0.05 or less was considered to indicate statistical significance.

## 3. Results

Thirty-six subjects with an average age of 48 ± 5 years recruited for the prospective two-arm longitudinal crossed-over intervention trial with regular moderate intake of traditional and alcohol-free beers completed both intervention phases and were included in the final analysis (Figure S1). Serum from these individuals, obtained before and after intervention with alcohol-free and traditional beer, was used to challenge the inflammasome pathway in induced macrophages, according to the methodology presented in the Methods section.

### 3.1. Effect of Beer Consumption on Cytokine Expression in Induced Macrophages (Signal 1)

**Figure 3:**
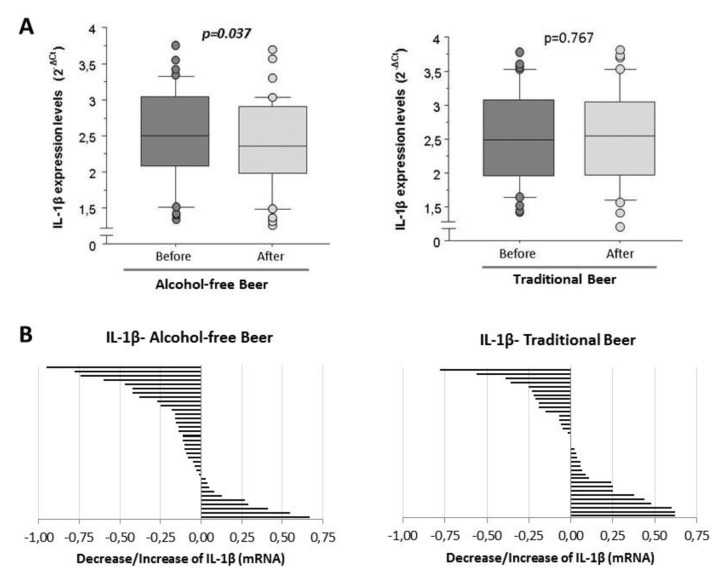
IL-1β expression levels in macrophages exposed to LPS in the presence of serum from healthy volunteers obtained before and after regular and moderate consumption of (**A**) Alcohol-free beer and Traditional beer for a period of four weeks. The results are expressed as median and quartiles 1–3 (horizontal lines). RT-qPCR values were normalized with the endogenous PIK3C2A gene by calibration curve. The *t*-student’s for paired samples of *p* < 0.05 are considered significant and refer to *n* = 36 individuals analyzed independently; (**B**) changes in the individual expression levels of IL-1β mRNA in macrophages. Effects induced at the individual level (*n* = 36) by blood serum after intervention with alcohol-free beer and traditional beer.

Human THP-1 cells were differentiated into macrophages by incubation with Phorbol 12-myristate 13-acetate (PMA) (Figure S2) [24]. Human macrophages induced with LPS show mean expression levels of 2.51 ± 0.08 (2^−ct^) (range from 1.33 to 3.78 in RT-qPCR normalized with the endogenous PIK3C2A gene by calibration curve) when they were conditioned with human serum obtained before the intervention (baseline). Macrophages conditioned with human serum obtained after four weeks of moderate and daily regular intake of alcohol-free beer showed a significantly lower induction of IL-1β mRNA transcripts (*n* = 36). This effect was not evident after the consumption of traditional beer (*n* = 36; Figure 3A).

As shown in Figure 3B, in 72% of the individuals included in the study, the serum obtained after the ingestion of alcohol-free beer conditioned macrophages to a lower transcription of IL-1β than that of the same individuals before beer consumption. This effect was less evident after the consumption of traditional beer (favorable response with serum from 44% of the participants in the intervention study), with a statistically significant difference in the response between both groups (72% vs. 44%, *p* = 0.017 Chi-square test).

The mRNA levels of IL-18 and IL-6 did not show statistically significant differences in macrophages conditioned with serum obtained before and after the beer intake intervention in neither alcohol-free nor traditional beer.

**Figure 4:**
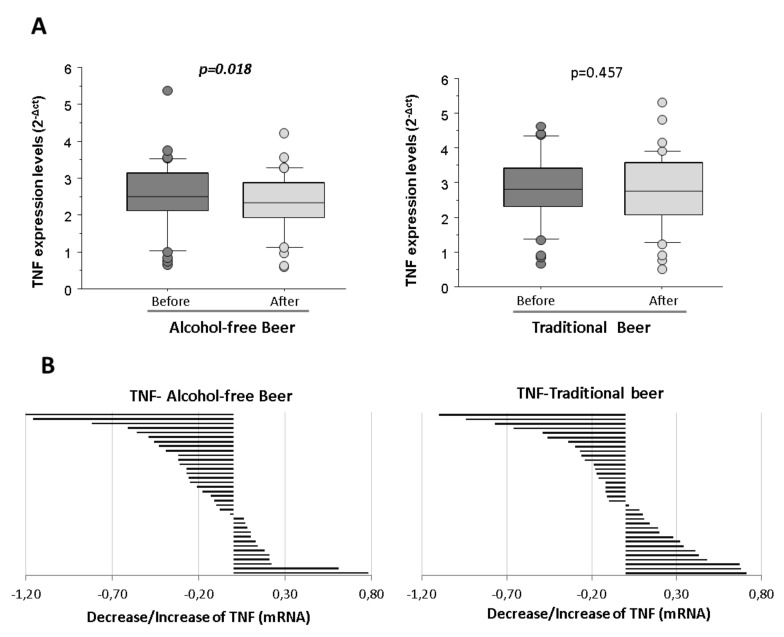
Expression levels of TNF in macrophages exposed to LPS in the presence of serum from healthy volunteers obtained before and after regular and moderate consumption of (**A**) alcohol-free beer and traditional beer for a period of four weeks. The results are expressed as median and quartile 1–3 (horizontal lines). RT-qPCR analyses were normalized with the endogenous PIK3C2A gene by calibration curve. The Student *t*-values for paired samples *p* < 0.05 are considered significant and refer to *n* = 36 individuals analyzed independently; (**B**) changes in the individual expression levels of TNF mRNA in macrophages. Effects induced at the individual level (*n* = 36) by blood serum after intervention with alcohol-free beer and traditional beer.

Macrophages stimulated with LPS and conditioned with human serum obtained after the intervention of alcohol-free beer exhibited a statistically significant lower transcription of TNF than when conditioned with serum obtained before beer consumption (*p* = 0.018). On the contrary, the decrease in the expression of TNF at the macrophage level was not observed after the consumption of traditional beer (Figure 4A).

The reduction was observed in the serum from 64% of the individuals that participated in the intervention study after the alcohol-free beer intervention, whereas the reduction after traditional beer referred to 55% of the volunteers (Figure 4B).

No differences were observed when sex (as a variable) was considered in the analysis for the different interleukins. However, only women showed a significant increase of IL-6 after the intake of traditional beer (Table S2).

As shown in Table 1, the changes induced by beer consumption on the cellular inflammasome pathway molecules show a similar pattern of response for IL-1β, TNF, and IL-6, while this pattern was less consistent for IL-18. Correlation analysis shows that the response of the different variables analysed is more homogeneous after the consumption of alcohol-free beer than traditional beer.

### 3.2. Effect of Human Serum on NF-κB and Caspase-8 Expression Levels in LPS-Stimulated Macrophages after Four Weeks of Moderate and Regular Beer Consumption

Macrophages conditioned with human serum obtained after four weeks of moderate and daily regular intake of alcohol-free beer did not induce significant changes of NF-κB transcript levels (2.13 ± 0.11 vs. 2.28 ± 0.19, *p* = 0.370), whereas a daily regular intake of traditional beer non-significantly decreased the mRNA (2^−Δct^) levels of NF-κB (2.34 ± 0.16 vs. 2.09 ± 0.12, *p* = 0.051).

Caspase-8 mRNA (2^−Δct^) was consistently expressed in conditioned macrophages with a mean value of 1.11 ± 0.02 at baseline. Compared to the expression of caspase-8 in basal conditions (serum before the beer intervention), the serum obtained after the intervention with alcohol-free beer induced a statistically non-significant reduction in the expression of caspase-8.

**Figure 5:**
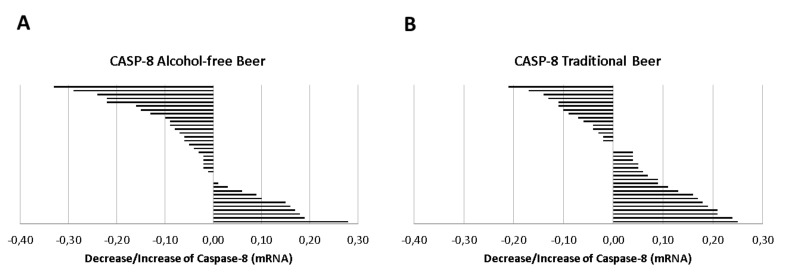
Distribution of the individual response (decrease/increase) of (**A**) alcohol-free beer or (**B**) traditional beer in the expression of Caspase-8 on macrophages exposed to LPS in the presence of serum obtained before and after regular and moderate beer consumption during a period of four weeks.

However, as shown in Figure 5A, in 64% of the individuals included in the study, the serum obtained after the ingestion of alcohol-free beer conditioned macrophages to a lower transcription of Caspase-8 than that of the same individuals before beer consumption. This effect was less evident after the consumption of traditional beer, Figure 5B (favorable response with serum from 42% of the participants in the intervention study), with a statistically significant difference in the response between both groups (64% vs. 42%, *p* = 0.050 Chi-square test).

Changes in NF-κB expression levels were not associated with those observed in the expression levels of IL-1β, IL-18, IL-6, and TNF after both alcohol-free and traditional beer interventions (*p* > 0.050).

**Figure 6:**
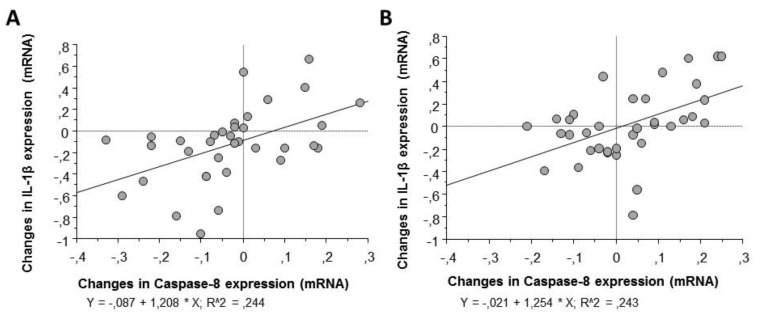
Linear regression for changes in caspase-8 and IL-1β gene expression in macrophages stimulated with LPS and exposed to serum obtained before and after regular and moderate consumption of (**A**) alcohol-free beer or (**B**) traditional beer.

In contrast, correlation analysis by Pearson’s test (Table 2) showed a positive association between the changes observed in the expression patterns of caspase-8 in macrophages and those observed in IL-1β, the final product of the inflammasome cascade, as well as with the other molecular intermediaries of the signaling process (Figure 6).

### 3.3. Effect of Human Serum after Four Weeks of Moderate and Regular Beer Consumption on NLRP3 and AIM2 Inflammasomes Expression Levels in LPS-Stimulated Macrophages

**Figure 7:**
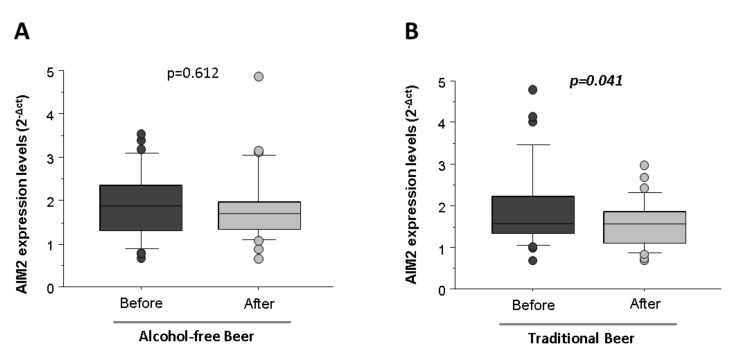
Expression levels of AIM2 in macrophages exposed to LPS in the presence of serum from healthy volunteers obtained before and after regular and moderate consumption of (**A**) alcohol-free beer and (**B**) traditional beer for a period of four weeks. The results are expressed as median and quartile 1–3 (horizontal lines). RT-qPCR analyses were normalized with the endogenous PIK3C2A gene by calibration curve. The Student *t*-values for paired samples *p* < 0.05 are considered significant and refer to *n* = 36 individuals analyzed independently.

Macrophages conditioned with human serum obtained after four weeks of moderate and daily regular intake of alcohol-free beer did not induce significant changes of NLRP3 (5.92 ± 0.20 vs. 5.92 ± 0.33, *p* = 0.984) and AIM2 (1.89 ± 0.14 vs. 1.82 ± 0.14, *p* = 0.612, Figure 7A) mRNA transcripts.

Nevertheless, a daily regular intake of traditional beer non-significantly decreased the mRNA (2^−Δct^) levels of NLRP3 (6.50 ± 0.56 vs. 5.99 ± 0.20, *p* = 0.314) and induced a statistically significant reduction in the expression of AIM2 inflammasome (1.89 ± 0.17 vs. 1.56 ± 0.10, *p* = 0.041, Figure 7B).

### 3.4. Effect of Volunteers Overweight and Obesity on Serum-Induced Cytokine Expression in Induced Macrophages

**Figure 8:**
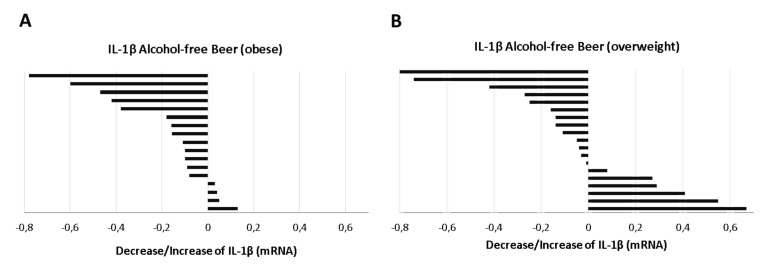
Distribution of the response (decrease/increase) of (**A**) obese or (**B**) overweight individuals in the expression of IL-1β on macrophages exposed to LPS in the presence of serum obtained before and after regular and moderate consumption of alcohol-free beer during a period of four weeks.

The serum-induced expression of IL-1β mRNA in macrophages did not show significant differences at baseline (time 0) between overweight (*n* = 19, BMI: 28.0–29.9 kg/m^2^) and obese individuals (*n* = 17, BMI: 30.0–35.0 kg/m^2^), (median [IQR]: IL-1β in the overweight group vs. obesity: 2.55 [3.16–1.82] vs. 2.48 [2.79–2.10], Mann–Whitney test *p* = 0.791). However, when the response to beer intervention was evaluated, it was found that the reduction on IL-1β mRNA levels induced by serum after alcohol-free beer consumption was more frequent in the obese group. Figure 8A. Thus, the serum of 76% of the individuals with BMI > 30 kg/m^2^ (*n* = 17) inhibit LPS stimulation of the inflammosome (*p* = 0.005, Student’s *t*-test for paired samples), while this effect was only observed in 68% (*n* = 19) of individuals with a BMI between 28.0 and 29.9 kg/m^2^ (Figure 8B).

The effect of alcohol-free beer intake on IL-1 β expression in obese volunteers was evident in both men (*p* = 0.035) and women, although in the latter the difference from the baseline value did not reach statistical significance (*p* = 0.089), possibly due to limited population size (*n* = 7).

On the contrary to alcohol-free beer, the consumption of traditional beer did not produce any effect on the expression of IL-1β in macrophages when separately analyzing the overweight and/or obesity groups (*p* = 0.445 and *p* = 0.672, respectively, Student’s *t*-test for paired samples).

IL-18 and IL-6 expression did not show significant differences by conditioning with serum from volunteers before and after beer consumption when overweight and obese individuals were analyzed separately.

**Figure 9:**
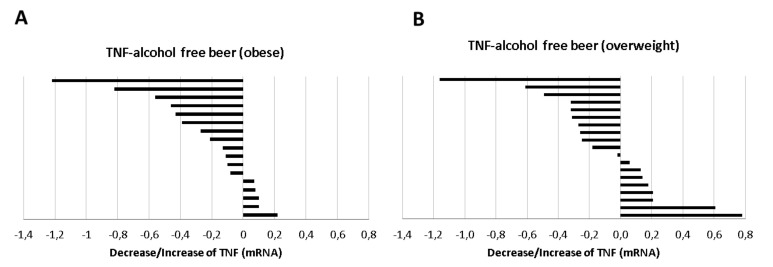
Distribution of the response (decrease/increase) of (**A**) obese or (**B**) overweight individuals in the expression of TNF on macrophages exposed to LPS in the presence of serum obtained before and after regular and moderate consumption of alcohol-free beer during a period of four weeks.

Induced-macrophages conditioned with human serum of obese individuals after beer ingestion period showed a lower TNF expression that reached statistical significance (−0.217 ± 0.090, *p* = 0.006 regardless of the type of beer ingested, an effect that was not observed in the subgroup with IMT <30.0 kg/m^2^. The decrease in TNF in the obese population was especially evident after the consumption of alcohol-free beer (−0.373 ± 0.090; *p* = 0.014, Figure 9A, with a positive response from 70.5% the serum of obese individuals whereas the positive response was 58% in the overweight volunteers, Figure 9B. This effect, on the other hand, was not observed with traditional beer.

### 3.5. Effect of Beer Consumption on IL-1β Protein Levels in Primed and Activated THP-1 Macrophages

**Figure 10:**
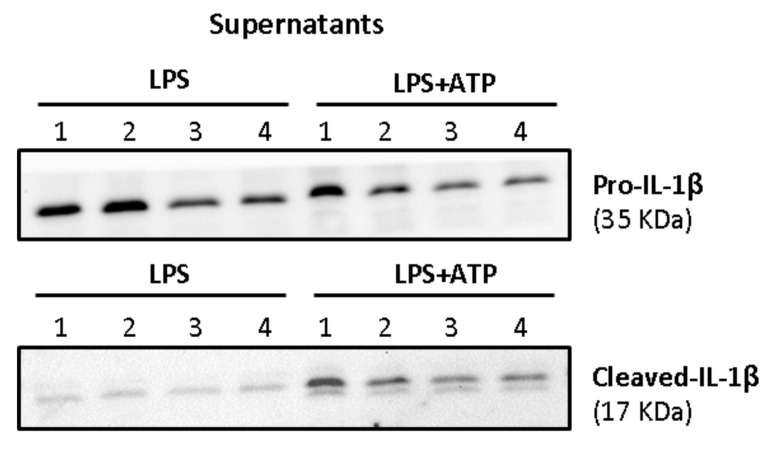
Western blot showing the secreted forms of IL-1β in supernatants after priming (LPS) or activation (ATP in LPS stimulated cells) of THP-1 macrophages. The 35 kDa band refers to pro-IL-1β and the 17 kDa band to its mature form (Cleaved-IL-1β). Lanes refer to the four interventions of one representative volunteer: lanes 1 and 2 (alcohol-free beer; before and after), lanes 3 and 4 (traditional beer; before and after).

Activated macrophages release large amounts of mature IL-1β, the biological active form. However, a signal 2 (ATP-dependent) is required for the activation of the inflammasome complex. As shown in Figure 10, the treatment with only LPS was not capable of inducing the release of cleaved-IL-1β (mature IL-1β). LPS induced increased protein levels of pro-IL-1β in the supernatants, but only the mature form of the interleukin was consistently observed in ATP-treated macrophages.

Therefore, protein forms of IL-1β were studied after both priming and activation signals. To this aim, individuals (*n* = 10) exhibiting the best responses in the mRNA expression levels were investigated to assess whether the changes observed in gene expression were reflected at protein level.

**Figure 11:**
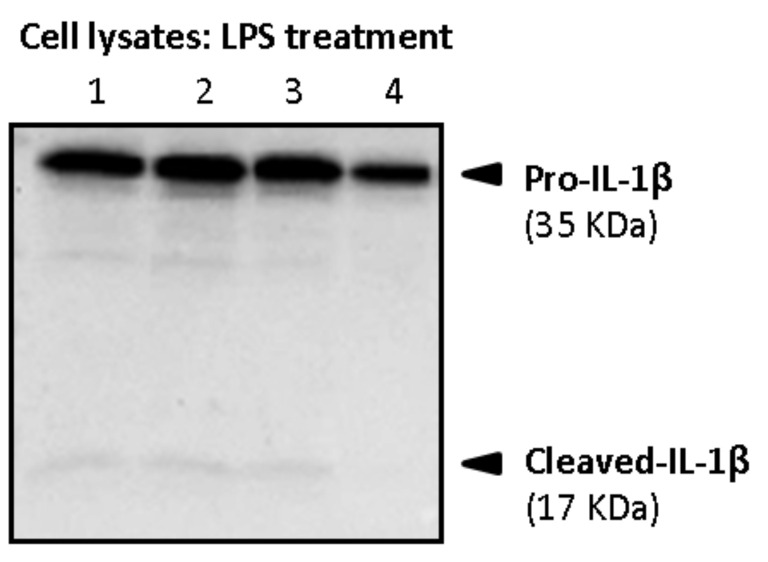
Western blot showing induction of intracellular 35 kDa pro-IL-1β and 17 kDa (Cleaved-IL-1β) after stimulation of macrophages with LPS. Lanes refer to the four interventions of one representative volunteer: lanes 1 and 2 (alcohol-free beer; before and after), lanes 3 and 4 (traditional beer; before and after).

Firstly, cell lysates and supernatants were analyzed for the determination of both pro-IL-1β and mature forms in primed macrophages. As shown in Figure 11, intracellular pro-IL-1β in primed macrophages mainly referred to the precursor form of 35 kDa (pro-IL-1β), whereas little intracellular amounts of its mature form (cleaved-IL-1β, 17 kDa) were detected.

**Figure 12:**
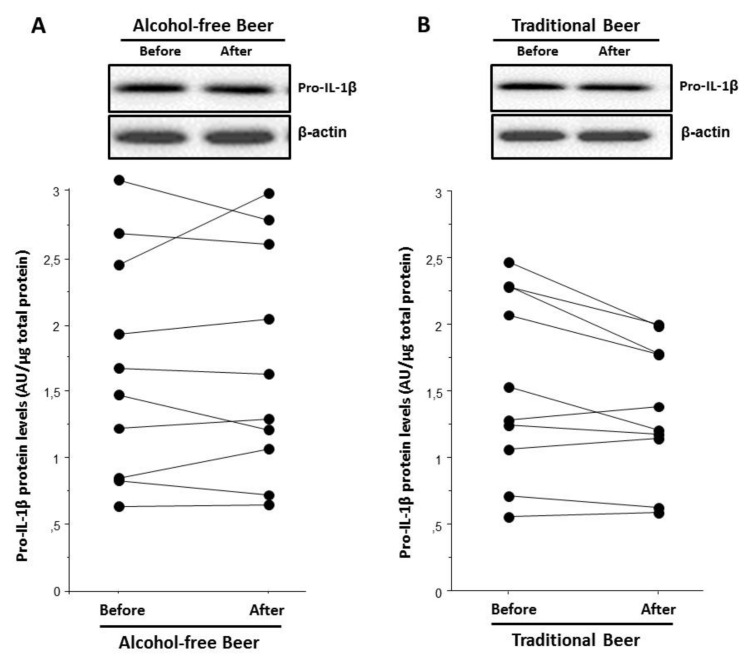
Individual changes of Pro-IL-1β intracellular protein levels of macrophages exposed to LPS in the presence of serum obtained before and after a moderate consumption of (**A**) alcohol-free beer or (**B**) traditional beer during a period of four weeks.

The assessment of serums from the beer interventional study showed that the serum after traditional beer intake induced a lower intracellular pro-IL-1β protein expression level than the serum before beer intake (*p* = 0.029, Figure 12B). This was not observed after alcohol-free beer, Figure 12A.

**Figure 13:**
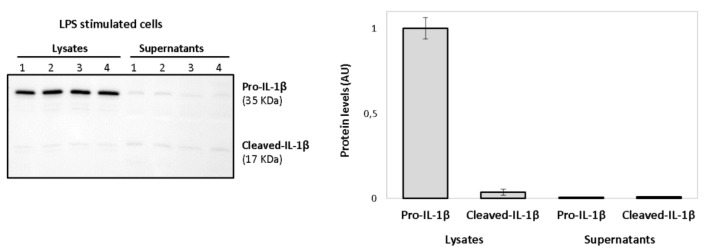
Western blot showing induction of intracellular 35 kDa pro-IL-1β after LPS treatment of macrophages. The secreted 17 kDa form (Cleaved-IL-1β) was weakly induced after LPS stimulation either in the supernatants or at intracellular level. Lanes refer to the four interventions of one representative volunteer: lanes 1 and 2 (alcohol-free beer; before and after), lanes 3 and 4 (traditional beer; before and after).

In LPS-stimulated cells, little amounts of pro-IL-1β and its mature form were detected in the supernatants when compared to the cell lysates, as shown in Figure 13. No statistically significant differences were observed in supernatants’ values.

Lastly, both pro-IL-1β and mature IL-1β were analyzed in the supernatants of ATP-activated macrophages. Results (Table 3) showed that the treatment of macrophages with the serum obtained before and after both interventions did not induce changes in the protein levels of pro-IL-1β. Nevertheless, treatment after traditional beer induced a decrease (*p* = 0.045) in the protein levels of the mature IL-1β that was only significant when results were expressed as the levels of mature IL-1β released respect to the total (pro-IL-1β + cleaved-IL-1β).

## 4. Discussion

Consistent epidemiological evidence indicates that low to moderate alcohol consumption in the form of fermented beverages is inversely correlated with adverse cardiovascular outcomes, conferring protective effects against overall mortality, mainly from coronary heart disease. However, excessive alcohol intake is associated with increased cardiovascular risk [25]. Low to moderate doses of alcohol are related to decreased inflammatory markers such as the C-reactive protein (CRP) and certain cytokines (IL-6 and TNF) [26], while high doses promote oxidative stress and a wide variety of inflammatory markers [27].

Fermented beverages, such as beer, would confer a greater cardiovascular protection than spirits, which have much less polyphenol content. A previous study performed with beer showed that alcohol fraction improved the lipid profile and reduced some plasma inflammatory biomarkers related to atherosclerosis, while the polyphenol content reduced leukocyte adhesion and inflammatory molecules [28]. Polyphenols have been described to interfere in multiple anti-inflammatory pathways such as the NF-kβ, which is necessary for the proper activation of the NLRP3 inflammasome, repressing the expression of TNF, IL-6, and IL-1β, which are linked to CVD [29,30,31].

Scientific evidence in relation to the effects derived from beer consumption on inflammatory/anti-inflammatory responses is scarce. Thus, we provide first time evidence on the capacity of beer intake, either alcohol-free or traditional, to modulate TLR-mediated inflammatory responses on induced macrophages.

The results of this study showed that induced macrophages conditioned with serum obtained after the ingestion of alcohol-free beer presented a lower transcription of different pro-inflammatory interleukins such as IL-1β and TNF compared to serum from the same individuals before beer consumption. Interestingly, the levels of these pro-inflammatory interleukins decreased despite the levels of NF-κB transcripts not showing significant changes. However, it is believed that caspase-8 regulates activation of NF-κB to modulate inflammation. Evidence suggests that caspase-8 is required for optimal expression of pro-IL-1β. In response to TLR4 stimulation, caspase-8 is recruited to a complex containing IKKαβ, and its loss results in delayed NF-κB nuclear translocation [32,33]. In this study, 64% of the individuals reduced the transcript levels of Caspase-8 after the consumption of alcohol-free beer, which, in turn, may delay the translocation of NF-κB to the nucleus producing decreased mRNA expression levels of NF-κB dependent cytokines such as pro-IL-1β and TNF. Furthermore, changes in the levels of Caspase-8 were strongly associated with changes in pro-inflammatory interleukins such as pro-IL-1β, TNF, and IL-6, strengthening the idea of a regulatory role for Caspase-8 on interleukin expression through NF-κB modulation.

Hence, alcohol-free beer exhibited the capacity to attenuate the inflammatory response once macrophages are induced to activation. However, this effect was not observed with traditional beer.

The capacity of beer to modulate the priming signal on macrophages was studied at the protein level, the only serum obtained after traditional beer intake significantly decreased intracellular protein levels of pro-IL-1β albeit selected individuals in this substudy showed the best responses for both interventions at the transcriptional level. Furthermore, when inflammasome activation was induced by ATP, a similar effect was observed and only serum after traditional beer consumption significantly diminished the protein release of the mature form of IL-1β (cleaved-IL-1β) to the supernatants. In our study, we observed that LPS also potentiates AIM2 inflammasome expression. A previous study suggested that, in addition to increasing NLRP3 inflammasome activity via NLRP3 induction, LPS boosts caspase-1 activation by the NLRP3 and AIM2 inflammasomes by an acute mechanism that is independent of inflammasome sensor induction [34]. In addition, another study shows that activation of AIM2 and NLRP3 provides redundant roles in mediating IL-1β release and that both induce a single cytoplasmic inflammasome platform [35]. Therefore, though NLRP3 expression levels remained unchanged we observed that AIM2 levels decreased significantly after the intake of traditional beer, which may explain the significantly reduced protein release of the mature form of IL-1β.

Surprisingly, although a greater anti-inflammatory effect would be expected in traditional beer due to the combination of the alcoholic and phenolic fraction, certain studies suggest that polyphenols would have a regulatory role in the priming pathway of the inflammasome, while alcohol would have an effector role in its activation pathway, modulating inflammatory responses at the protein level. For instance, in a mice model of stress and depression, both conditions linked to a pro-inflammatory state, polyphenols were capable of attenuating the chronic stress state of the mice by inhibiting neuronal cell priming, which decreased neuroinflammatory status [18]. Moreover, polyphenols exhibited the capacity to suppress tumor growth by reducing NF-kβ activity and IL-1β secretion [36]. Other studies provide evidence that the alcoholic fraction (ethanol) inhibits the activation of the NLRP3 and AIM2 inflammasomes [19,37,38]. The different metabolites derived from the hydrolysis of alcohol seem to specifically inhibit the NLRP3 inflammasome by preventing potassium efflux and ASC oligomerization. Furthermore, short chain alcohols would exert an inhibition of the NLRP3 inflammasome by decreasing the overall tyrosine phosphorylation.

In the present study, we found a decreased induction of IL-1β and TNF when macrophages were conditioned with the serum of obese individuals compared to those with overweight. One of the reasons why obese individuals may present a better response to both interventions might be related to the fact that obesity is linked to a higher oxidative and inflammatory state. We had previously shown that the obese individuals have a higher Framingham risk score than overweight subjects and have higher systemic levels of CRP and TNF though differences do not reach statistical significance because all of them are healthy individuals without other CVRF [22]. Therefore, it is possible that beer uptake had a stronger effect on obese individuals compared to those individuals with less cardiovascular risk.

The fact that traditional beer, despite containing polyphenols, has not shown the same ability to modulate inflammatory responses could be due to a possible interaction of polyphenols and the alcoholic fraction in the priming pathway.

The current study has some limitations that are worthy of discussion. Although this study is based on a well-characterized cohort of healthy but overweight/obese individuals, it has a small sample size that is not representative of a more general population. However, the longitudinal cross-over design gives strength to the results of the study minimizing inter-individual variability for the effects observed with the traditional and alcohol-free beer. Furthermore, due to the short duration of the intervention for each beer type, our results might not reflect the potential risks/benefits of longer-term moderate beer consumption related to inflammatory responses.

## 5. Conclusions

The present study shows that regular moderate intake of traditional and alcohol-free beer attenuates the inflammasome signalling pathway in human macrophages. In particular, a moderate intake of alcohol-free beer was associated with a lower transcription of different pro-inflammatory interleukins, while the serum obtained after the consumption of traditional beer reduced protein release of the active mature form of IL-1β. These hypothesis generating results encourage further studies with larger sample size and extended intervention periods in order to better assess the long-term balance of benefits/risks of moderate beer intake in cardiovascular health.

## Figures and Tables

**Table 1:** Correlations between variables calculated using the Pearson test (correlation value [lower 95%/higher 95%]. The response for each variable refers to the difference between the value at the end and at the beginning of the intervention with traditional beer or without alcohol. *n* = 36 (total of the study population).

Alcohol-Free Beer	IL-1β	IL-18	TNF	IL-6
IL-1β		0.447 [0.139/0.676] *p* = 0.006	0.621 [0.367/0.789] *p* < 0.001	0.654 [0.414/0.809] *p* < 0.001
IL-18			0.351 [0.025/0.609] *p* = 0.035	0.482 [0.182/0.700] *p* = 0.002
TNF				0.503 [0.209/0.714] *p* = 0.002
**Traditional Beer**	**IL-1** **β**	**IL-18**	**TNF**	**IL-6**
IL-1β		0.032 [−0.300/0.356] *p* = 0.856	0.388 [0.068/0.636] *p* = 0.019	0.541 [0.258/0.738] *p* < 0.001
IL-18			0.338 [0.011/0.600] *p* = 0.043	0.361 [0.037/0.616] *p* = 0.030
TNF				0.426 [0.113/0.662] *p* = *0.010*

**Table 2:** Correlations between variables calculated using the Pearson test (correlation value [lower 95%/higher 95%]. The response for each variable refers to the difference between the value at the end and at the beginning of the intervention with alcohol-free beer or traditional beer. *n* = 36 (total of the study population).

	NF-κB	IL-1β	IL-18	TNF	IL-6
**Alcohol-Free Beer**					
**Caspase-8**	0.434 [0.094/0.683] *p* = 0.014	0.494 [0.198/0.708] *p* = 0.002	0.320 [−0.009/0.587] *p* = 0.056	0.402 [0.084/0.645] *p* = 0.014	0.554 [0.263/0.741] *p* < 0.001
**Traditional Beer**					
**Caspase-8**	0.452 [0.129/0.689] *p* = 0.008	0.493 [0.196/0.707] *p* = 0.002	0.111 [−0.226/0.424] *p* = 0.522	0.427 [0.114/0.663] *p* = 0.009	0.536 [0.252/0.735] *p* < 0.001

**Table 3:** Changes of Pro-IL-1β and Cleaved-IL-1β protein levels in the supernatants of macrophages exposed to ATP in the presence of serum obtained before and after a moderate consumption of alcohol-free beer or traditional beer during a period of four weeks. Values were normalized by the total amount of protein (Ponceau) and given as the mean ± SEM. Percentage of released IL-1β values were expressed as the levels of mature IL-1β released respect to the total (pro-IL-1β + cleaved-IL-1β).

	Alcohol-Free Beer		*p*-Value	Traditional Beer		*p*-Value
	Before	After		Before	After	
**Pro-IL-1 β (N = 8)**	1.06 ± 0.2	1.20 ± 0.5	0.435	0.94 ± 0.2	1.03 ± 0.3	0.370
**Cleaved-IL-1 β (N = 8)**	1.58 ± 1.9	2.13 ± 3.1	0.424	1.46 ± 1.5	1.23 ± 1.1	0.418
**% Released IL-1 β (N = 8)**	36.6 ± 10.9	35.4 ± 11.3	0.506	40.3 ± 11.0	37.6 ± 10.4	0.045

## Supplementary Materials

The following are available online at https://www.mdpi.com/article/10.3390/biology10111159/s1, Figure S1: Study Design; Figure S2: Characterization of THP-1 macrophages through the gene expression levels of different markers associated with monocyte-macrophage differentiation (PMA), Table S1: Assay IDs for quantitative real-time (QRT) PCR, Table S2: Expression levels in regard to sex of different interleukins in macrophages exposed to LPS in the presence of serum from healthy volunteers obtained before and after regular and moderate consumption of alcohol-free beer and traditional beer for a period of 4 weeks, and full Western Blot images.
