# Supplementary material for: Moderate Beer Intake Downregulates Inflammasome Pathway Gene Expression in Human Macrophages
Source: Biology (Basel). 2021 Nov 9;10(11):1159. doi: 10.3390/biology10111159 (PMC8614923; doi:10.3390/biology10111159)
Supplement: Supplementary file 1 [file biology-10-01159-s001.zip › biology-1452439-suppl.pdf]

Supplementary Material

Moderate Beer Intake Downregulates Inflammasome Pathway Gene Expression in Human Macrophages

Natalia Muñoz-García[1], Rafael Escate[2], Lina Badimon [1,2,3*], Teresa Padro[1,2]

[1] Cardiovascular Program-ICCC, Research Institute- Hospital Santa Creu i Sant Pau, IIB-Sant Pau, Barcelona.Spain
[2] Centro de Investigación Biomédica en Red cardiovascular (CIBERCV) Instituto de Salud Carlos III, Madrid, Spain.
[3]Cardiovascular Research Chair, UAB, Barcelona, Spain

Address for corresponding author (*):

Prof. Lina Badimon
Cardiovascular Program-ICCC
Research Institute Hospital Santa Creu i Sant Pau
Sant Antoni Mª Claret 167, 08025 Barcelona, Spain
Phone: +34 935565886
Fax: +34 935565559
E-mail: lbadimon@santpau.cat

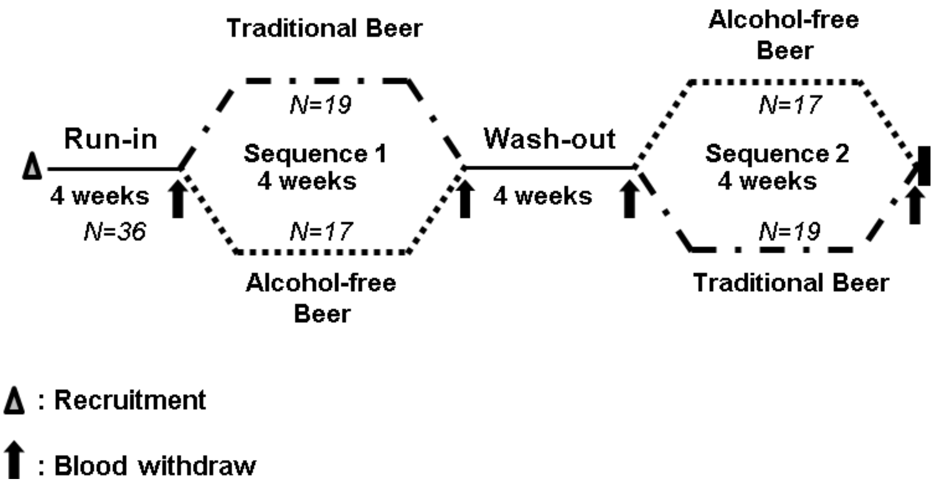

Figure S1. Peripheral blood (serum) samples were obtained from healthy adult individuals submitted to two consecutive intervention periods (4 weeks) with alcohol-free beer (660 ml men and 330 ml women / day, 0g alcohol per day) or traditional beer (660 ml men and 330 ml women / day corresponding to 30g alcohol / day and 15g alcohol / day, respectively). Modified from [22].

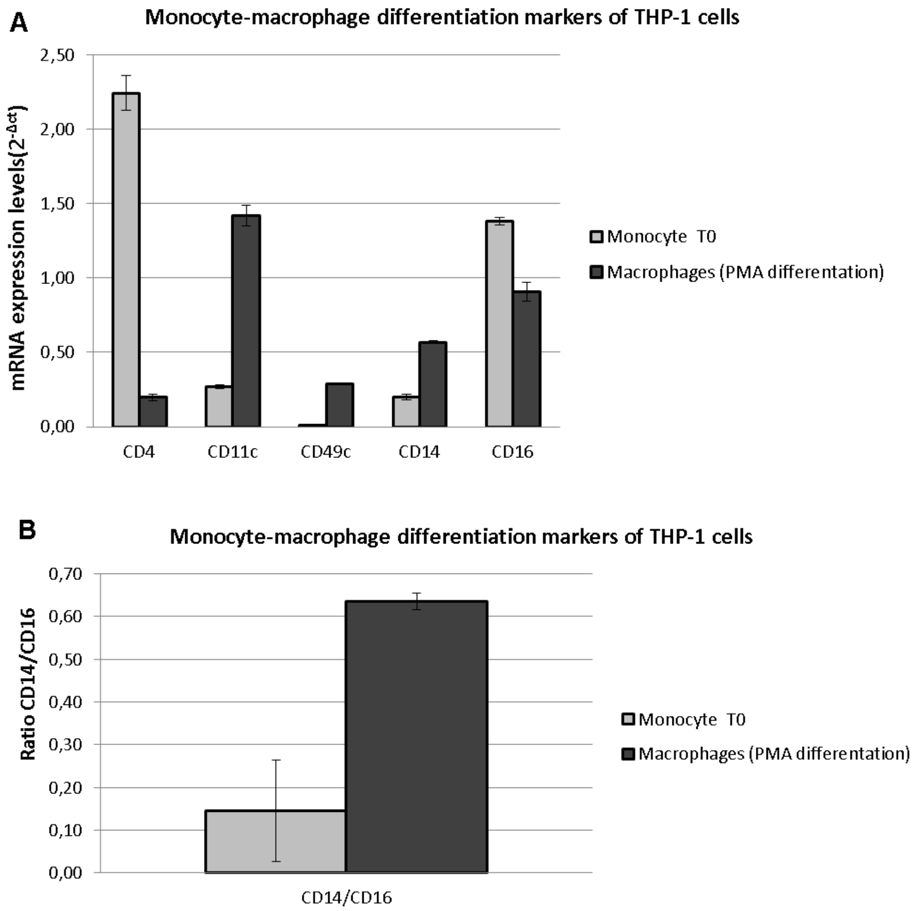

Figure S2A. Characterization of THP-1 macrophages through the gene expression levels of different markers associated to monocyte-macrophage differentiation (PMA):
CD4: Marker of THP-1 monocytes. Decreased in macrophages.
CD11c: Adhesion molecule during monocyte-macrophage differentiation. Increases during differentiation.
CD49c: Integrin increased during monocyte-macrophage differentiation.
CD14: Marker that increases during macrophage differentiation of THP-1 cells.
CD16: Less expressed in THP-1 macrophages.
Figure S2B. Ratio CD14/CD16: Increases showing a M1-like profile for THP-1 differentiated macrophages.

Table S1. Assay IDs for quantitative real-time (QRT) PCR.

Assay ID	Gene ID	Genomic map
**Hs01555410_m1**	IL1β	https://www.thermofisher.com/order/genome-database/details/gene-expression/Hs01555413_m1
**Hs01038788_m1**	IL18	https://www.thermofisher.com/order/genome-database/details/gene-expression/Hs01038788_m1
**Hs00174131_m1**	IL6	https://www.thermofisher.com/order/genome-database/details/gene-expression/Hs00174131_m1
**Hs00174128_m1**	TNF	https://www.thermofisher.com/order/genome-database/details/gene-expression/Hs00174128_m1
**Hs01022438_m1**	CASP8	https://www.thermofisher.com/order/genome-database/details/gene-expression/Hs01022438_m1
**Hs00231653_m1**	NF-κB	https://www.thermofisher.com/order/genome-database/details/gene-expression/Hs00231653_m1
**Hs00918082_m1**	NLRP3	https://www.thermofisher.com/order/genome-database/details/gene-expression/Hs00918082_m1
**Hs00175457_m1**	AIM2	https://www.thermofisher.com/order/genome-database/details/gene-expression/Hs00175457_m1
**Hs00153223_m1**	PIK3C2A	https://www.thermofisher.com/order/genome-database/details/gene-expression/Hs00153223_m1
**Hs01058407_m1**	CD4	https://www.thermofisher.com/order/genome-database/details/gene-expression/Hs01058407_m1
**Hs00174217_m1**	CD11c	https://www.thermofisher.com/order/genome-database/details/gene-expression/Hs00174217_m1
**Hs00233722_m1**	CD49c	https://www.thermofisher.com/order/genome-database/details/gene-expression/Hs00233722_m1
**Hs00169122-g1**	CD14	Not available
**Hs00275547_m1**	CD16	https://www.thermofisher.com/order/genome-database/details/gene-expression/Hs00275547_m1

Table S2. Expression levels in regard to sex of different interleukins in macrophages exposed to LPS in the presence of serum from healthy volunteers obtained before and after regular and moderate consumption of alcohol-free beer and traditional beer for a period of 4 weeks.

	**Alcohol-free Beer**		**p-value**	**Traditional Beer**		**p-value**
	Before	After		Before	After	
**IL-1β**						
Men (N=21)	2.32±0.1	2.22±0.1	0.081	2.45±0.1	2.42±0.1	0.570
Women (N=15)	2.73±0.1	2.58±0.2	0.209	2.65±0.1	2.73±0.1	0.473
**IL-18**						
Men (N=21)	0.68±0.1	0.70±0.1	0.633	0.71±0.1	0.73±0.1	0.457
Women (N=15)	0.52±0.04	0.51±0.04	0.550	0.53±0.05	0.59±0.05	0.062
**TNFα**						
Men (N=21)	2.53±0.2	2.34±0.2	0.060	2.95±0.2	2.80±0.2	0.131
Women (N=15)	2.47±0.3	2.33±0.2	0.179	2.66±0.3	2.75±0.3	0.368
**IL-6**						
Men (N=21)	2.50±0.2	2.50±0.1	0.968	2.73±0.2	2.69±0.2	0.714
Women (N=15)	2.80±0.3	2.74±0.2	0.704	2.71±0.3	3.04±0.3	***0.018***

Results are expressed as the mean ± SE. Student t values for paired samples $p < 0.05$ are considered significant.
